# *Candida albicans* Increases Tumor Cell Adhesion to Endothelial Cells *In Vitro*: Intraspecific Differences and Importance of the Mannose Receptor

**DOI:** 10.1371/journal.pone.0053584

**Published:** 2013-01-02

**Authors:** Andoni Ramirez-Garcia, Beatriz Arteta, Ana Abad-Diaz-de-Cerio, Aize Pellon, Aitziber Antoran, Joana Marquez, Aitor Rementeria, Fernando L. Hernando

**Affiliations:** 1 Department of Immunology, Microbiology and Parasitology, Faculty of Science and Technology, University of the Basque Country (UPV/EHU), Leioa, Spain; 2 Department of Immunology, Microbiology and Parasitology, Faculty of Pharmacy, University of the Basque Country (UPV/EHU), Vitoria-Gasteiz, Spain; 3 Department of Cell Biology and Histology, Faculty of Medicine and Dentistry, University of the Basque Country (UPV/EHU), Leioa, Spain; Charité-University Medicine Berlin, Germany

## Abstract

The dimorphic fungus *Candida albicans* is able to trigger a cytokine-mediated pro-inflammatory response that increases tumor cell adhesion to hepatic endothelium and metastasis. To check the intraspecific differences in this effect, we used an *in vitro* murine model of hepatic response against *C. albicans*, which made clear that tumor cells adhered more to endothelium incubated with blastoconidia, both live and killed, than germ tubes. This finding was related to the higher carbohydrate/protein ratio found in blastoconidia. In fact, destruction of mannose ligand residues on the cell surface by metaperiodate treatment significantly reduced tumor cell adhesion induced. Moreover, we also noticed that the effect of clinical strains was greater than that of the reference one. This finding could not be explained by the carbohydrate/protein data, but to explain these differences between strains, we analyzed the expression level of ten genes (*ADH1, APE3, IDH2, ENO1, FBA1, ILV5, PDI1, PGK1, QCR2* and *TUF1*) that code for the proteins identified previously in a mannoprotein-enriched pro-metastatic fraction of *C. albicans*. The results corroborated that their expression was higher in clinical strains than the reference one. To confirm the importance of the mannoprotein fraction, we also demonstrate that blocking the mannose receptor decreases the effect of *C. albicans* and its mannoproteins, inhibiting IL-18 synthesis and tumor cell adhesion increase by around 60%. These findings could be the first step towards a new treatment for solid organ cancers based on the role of the mannose receptor in *C. albicans*-induced tumor progression and metastasis.

## Introduction

*Candida albicans* is a dimorphic fungal species that is part of the normal human microbiota as a commensal and that does not usually cause infection. However, when the defense mechanisms of the host are impaired, it is capable of becoming pathogenic. In such situations, *C. albicans* is able to disseminate hematogenously, and to cause serious problems by spreading to multiple organs. On arrival to organs by via hematogenous, it first adheres to endothelial cells as blastoconidia or hyphal forms, although it has recently been demonstrated that there is greater adhesion with blastoconidia [1]–[4].

In the early stages of the adhesion, certain receptors such as the mannose receptor (MR), Toll-like receptors (TLRs), and dectin-1, play a crucial role in the recognition of evolutionarily conserved structures in the fungus [5], [6]. After adhesion, a pro-inflammatory response to *C. albicans* is initiated in which cytokines such as TNF-α, IL-1ß and IL-18 are involved [7]–[9]. This orchestrates the early host response to infection and, at the same time, represents an important link to the adaptive immune response by the activation and recruitment of many immune cells [10].

A few years ago, it was established that inflammation and cancer are related [11]. In addition, in previous papers, we have reported that the adhesion of *C. albicans* to hepatic sinusoidal endothelial (HSE) cells triggers a pro-inflammatory cytokine-mediated immune response, which contributes to the adhesion of B16 melanoma (B16M) cells and metastasis [12] by increasing the expression of the vascular cell adhesion molecule-1 (VCAM-1) [13]. This effect was also detected in a mannoprotein-enriched fraction (MPF) of a *C. albicans* clinical strain using an *in vitro* organ-specific model of hepatic microvascular response [14]. The pro-metastatic MPF in the blastoconidia of the strain was found to have a complex composition. Specifically, by proteomic techniques we identified the following proteins: Act1p, Adh1p, Ape3p, Dor14p, Eno1p, Fba1p, Idh2p, Ilv5p, Mpg1p, Pdi1p, Pgk1p, Qcr2p, Sfa1p, and Tuf1p [14]. Some of these could be related to the effect studied.

Accordingly, in patients suffering from cancer, the activation of endothelial cells either by *C. albicans* or its mannoproteins may be a risk factor for developing liver metastasis. The understanding of the mechanism by which the fungus enhances tumor cell adhesion may contribute to identify a suitable prophylaxis or treatment.

Consequently, the aims of this research were to study the effect of the morphology and viability of different strains of *C. albicans* on the increase in tumor cell adhesion to the endothelium, the importance of carbohydrate residues, and the expression of the proteins identified previously in the pro-metastatic MPF. Finally, we also tested the protective effect against the pro-metastatic process of a preventive blockade of the MR with a specific commercial antibody.

## Materials and Methods

### *C. albicans* strains, culture conditions and in vitro treatments

We used four well-characterized *C. albicans* strains in this study: UPV1413 (CECT13062) and UPV1360 (CECT13063) (both isolated from patients with systemic candidiasis and cryopreserved immediately); CA2 (a germination-deficient mutant, kindly provided by A. Cassone, Istituto Superiore di Sanità, Rome, Italy) and the reference strain NCPF3153. The use of the clinical strains from patients that had previously signed an anonymous informed consent form was approved by the Ethics Committee of the University of the Basque Country (UPV/EHU). Blastoconidia were obtained as previously described [14] and germ tubes by incubating 3×10^6^ blastoconidia/ml in RPMI medium (Sigma-Aldrich, St Louis, MO, USA) at 37°C for 4 h in a rotary shaker. To kill the cells, suspensions of 1×10^7^ *C. albicans* cells/ml were incubated at 70°C for 30 min in a water bath, or exposed to 20 mM or 50 mM sodium metaperiodate for 30 min at room temperature [8]. The metaperiodate treatment was also used to remove mannose residues. *C. albicans* cell viability was then assessed by culture.

The FACSCalibur flow cytometer (BD Biosciences, Franklin Lakes, NJ, USA) was used to check the presence of carbohydrates in *C. albicans*. For this, cells were incubated with 0.2 µg/ml of concanavalin A (Con A)-FITC, and data were analyzed using CellQuest software (V 3.3; BD Biosciences).

### Cultures of HSE and B16M cells

Syngeneic C57Bl/6J mice (males, 6–8 weeks old) were used. Samples were collected from these animals, and the HSE cells were isolated and purified by means of collagenase dissociation of the liver tissue, isopycnic centrifugation on a Percoll gradient and selective adherence, as follows. Mice were sacrificed by cervical dislocation, then their abdomen was cut open and a cannula inserted into the portal vein. The inferior vena cava was punctured to allow blood to drain out. Animals were perfused for 3 min (flow 7 ml/min, temperature 39°C), to flush the blood out of the liver. The liver tissue was then dissociated into single cell suspensions by perfusion with collagenase type IV from *Clostridium histolyticum* (Sigma-Aldrich). Following complete dissociation of the tissue, the resulting cell suspension was differentially centrifuged twice for 2 min at 30 g in 50-ml centrifuge tubes at 4°C yielding a parenchymal cell (PC)-enriched pellet and a non-PC-enriched supernatant. The PC-depleted supernatant was layered on top of a two-step Percoll gradient (25% on top of 50%) and subsequently centrifuged at 800× g for 30 min at 4°C. The interphase between the two density cushions, containing non-parenchymal cells (NPC)-enriched cell suspension, was collected. This fraction was diluted with RPMI 1640 medium and centrifuged (4°C; 800× g; 10 min). The resulting pellet was resuspended in RPMI 1640 medium without serum, and the cells were cultured twice for 10 min in a 9 cm Ø Petri dish to eliminate Kupffer cells by selective adherence. Then, non-adhered HSE cells were collected and the culture medium was supplemented with antibiotics and 5% fetal calf serum for culture on collagen type I (Sigma-Aldrich) covered tissue culture plates at a 0.3×10^6^ cells/cm^2^. After 45 min of culture, HSE cells were washed and allowed to spread for 2 h before being used for experiments. All incubations were carried out at 37°C in 5% CO_2_.

The viability was measured by the trypan blue exclusion test [15]. An aliquot containing 10 µl of HSE cell suspension was mixed with the same volume of trypan blue and the percentage of viable cells was calculated as follows: % viable cells  =  number of cells able to eliminate trypan blue dye/total number of cells. Cell suspensions containing a minimum of 90% of viable cells were used in the experiments. After treatments with *C. albicans* or MPF, the integrity of cells in monolayer culture was confirmed by microscopy. The experimental procedures involving the mice were approved by the Ethics Committee for animal welfare of the University of the Basque Country (UPV/EHU).

The mouse B16M cell line (CRL-6475) was obtained from the American Tissue Culture Collection (ATCC, Manassas, VA, USA). Cells were cultured and maintained as previously described [14].

### Analysis of IL-18 and tumor cell adhesion

The HSE cells (1×10^6^ cells/ml) were incubated with *C. albicans* (5×10^5^ cells/ml), MPF (2 µg carbohydrate/ml) or basal medium (pyrogen-free RPMI [Sigma-Aldrich] supplemented with 10% fetal bovine serum, 100 U/ml penicillin, and 100 μg/ml streptomycin) as control for 8 h. Next, the supernatants were collected and the IL-18 concentration measured using specific ELISA kits (R&D Systems, Minneapolis, MN, USA) according to the manufacturer's instructions. *C. albicans* yeast cells and germ tubes were then removed from the HSE cells by extensively washing.

After that, B16M cells were labeled with 2′,7′-bis-(2-carboxyethyl)-5,6-carboxyfluorescein-acetoxymethylester (BCECF-AM) (Invitrogen, Carlsbad, CA, USA) by incubation for 20 min. After washing in PBS by centrifugation, the cells were resuspended in fresh medium supplemented with antibiotics and added (2×10^5^ cells/well) to primary HSE culture. At this point, B16M cells were incubated for 4 min at 37°C and 5% CO_2_. Next, non-adherent cells were removed by aspiration and cultures were washed for three more times to eliminate non-adhered cells completely, before measuring the fluorescence using a cytofluorometric plate reader [13], [14].

The number of adhering cells was quantified in arbitrary fluorescence units based on the percentage relative to the initial number of tumor cells added to the HSE cells. Specifically, the percentages were calculated for each well as follows: % of adhered B16M cells  = 100× (fluorescence emission after washing – background fluorescence)/(total fluorescence emission before washing – background fluorescence), the background fluorescence being the natural fluorescence emitted by non-stained cells. Then, results for the HSE cells stimulated with *C. albicans* or mannoproteins were expressed in relative adhesion units compared to the control incubated with only medium.

Further experiments were conducted with anti-mouse mannose receptor antibody (anti-MR) (Acris antibodies, Hiddenhausen, Germany) added to the HSE cells for 30 min prior to the *C. albicans* cells or MPF. The suppressive effects of anti-MR were calculated as the percentage reductions in adhesion and IL-18 synthesis observed in assays when anti-MR was used, where a value of 100% was assigned to the mean obtained with each pro-inflammatory treatment without the use of anti-MR and 0% to the mean obtained in controls.

### Purified crude extracts and MPF of *C. albicans*

The crude extracts were obtained by sonication in lysis buffer [14] and the total protein and carbohydrate concentrations of the extracts were determined with an RC DC protein assay kit (Bio-Rad, Hercules, CA, USA) and the anthrone method [16], respectively, using bovine serum albumin or mannose as standards. The MPF of the UPV1413 strain was obtained as previously described [14]. Briefly, crude extracts were fractionated by Con A-Sepharose 4B affinity chromatography, and the MPF was eluted with 0.5 M methyl α-D-manno-pyranoside. Then, it was dialyzed against distilled water, lyophilized, and resuspended in endotoxin-free 50 mM phosphate-buffered saline (pH 7.4) (PBS) (Sigma-Aldrich). The endotoxins were removed as previously described [14], and the suspension was stored at −80°C.

### Gene expression analysis by quantitative real-time PCR

To synthetize cDNA, total RNA extracted with the Qiagen RNeasy Mini Kit (Qiagen Iberia, Madrid, Spain) was retrotranscribed to cDNA using the QuantiTect Reverse Transcription Kit (Qiagen), which eliminates genomic DNA before the quantitative real-time PCR (qPCR) reaction. For each sample, 1.8 µg of RNA were retrotranscribed in a total reaction volume of 20 μl. Primer pairs were designed for the mRNA sequences of genes obtained from NCBI GenBank database using the Primer Express software (version 3.0; Applied Biosystems, Foster City, CA, USA) and they were purchased from Invitrogen (Table 1).

**Table 1:** PCR primers used for the analysis of gene expression by quantitative real time PCR.

Genes	Gene name	NCBI number	Size (bp)	Primer sequence (5′ to 3′)
***ADH1***	Alcohol dehydrogenase	CaO19.3997	139	For: TTTAGCCAATGTCGCACCAA
				Rev: GGCGTATTGAACGGCCAAAG
***APE3***	Aminopeptidase Y	CaO19.3591	129	For: CGTGGTGAATGTGCCTTTGGT
				Rev: TGTGGCGACTTCTTTCCCAGTT
***ENO1***	Enolase	CaO19.395	133	For: AAACCCAGAATCCGACCCATC
				Rev: GACCCAAGCATCCCAGTCATC
***FBA1***	Fructose-bisphosphate aldolase	CaO19.4618	120	For: GCCAGAGACAACAAGGCTCCA
				Rev: GGCAGCAATTGAACCAGCAA
***IDH2***	Isocitrate dehydrogenase subunit	CaO19.5791	123	For: TGGGCCAGAAATTTCCCAAG
				Rev: GCTGGTTGTGGCAAGGTGGT
***ILV5***	Ketol-acid reductoisomerase	CaO19.88	131	For: CACGTTGAACCACCATCAAACA
				Rev: CCGGTAACATCGTTCCAGACA
***PDI1***	Disulfide isomerase	CaO19.5130	112	For: CCGTTGCCGATCCAAACA
				Rev: TAACCACACCATGGGGCAAA
***PGK1***	Phosphoglycerate kinase	CaO19.3651	131	For: GGCTTTGGAAAACCCAGAAAGA
				Rev: TGAAGGCCATACCACCACCA
***QCR2***	Ubiquinol-cytochrome-c reductase	CaO19.10167	127	For: TGGTGCATCTTCTGCTTTGATTG
				Rev: TGGAATTTGTGCTAATGGAGTGGA
***TUF1***	Translation elongation factor Tu	CaO19.6047	137	For: GCCCACGTTGAATACGAAACC
				Rev: CCATCAGTGGCAGCAACAACA
***ACT1***	Actin	CaO19.5007	111	For: GACGCTCCAAGAGCTGTTTTCC
				Rev: TTTGGATTGGGCTTCATCACC
***TDH3***	Glyceraldehyde 3- phosphate dehydrogenase	CaO19.6814	132	For: TGATGACCACTGTCCACTCCATC
				Rev: CAACGGCTTTAGCAGCACCA

The qPCR was performed on an ABI 7900HT Fast Real-Time PCR System using the Fast SYBR® Green Master Mix Kit (both from Applied Biosystems) and fast cycling conditions. Each sample and gene was run in triplicate to ensure statistical significance and 1 µl of diluted cDNA (4.5 ng/µl) was used per reaction (20 μl). We also analyzed a no template control, in which nuclease-free water was added instead of cDNA, and an additional no-RT (no retro-transcription) control, where no enzyme was added during cDNA synthesis reaction.

The reaction conditions were those established by the manufacturer and a dissociation stage was added after completion of amplification for melting curve analysis to determine the specificity of the PCR reaction.

A standard curve was generated with five serial dilutions of a concentrated cDNA sample generated from a pool of 10 samples. PCR amplification efficiency was calculated from the slopes of the standard curves, according to the equation E = −1+10^(−1/slope)^, where E stands for efficiency, and the slope is the gradient of the best fit line of the standard curve. Raw data were generated and processed with the Sequence Detection System (SDS) software (version 2.4; Applied Biosystems). GeNorm [17] and GenEx (version 5.3.5.6; MultiD Analyses AB, Göteborg, Sweden) software were employed to analyze the stability of the reference genes for normalization purposes, as well as to calculate the PCR efficiency correction. The relative expression levels were calculated by dividing the values obtained for each gene by the control values, taking the results for the *C. albicans* NCPF3153 blastoconidia as control in all cases.

### Statistical analysis

Data were expressed as means ± SD. Statistical analysis was performed using SPSS statistical software (version 6.0; Professional Statistic, Chicago, IL, USA). Homogeneity of the variance was tested using Levene's test. If the variances were homogeneous, data were analyzed using unpaired two-tailed Student's t-tests, while for data sets with non-homogeneous variances, the Mann-Whitney U test was used. The criterion for significance was set at *p*<0.05 for all comparisons.

## Results

### Effect of different strains and morphologies of *C. albicans* on tumor cell adhesion to HSE cells

It has been well known, for many years, that bloodstream clearance of *C. albicans* mainly occurs in the liver where large numbers of yeast cells accumulate [18] and, moreover, that this fungus stimulates an inflammatory response through the local expression of cytokines and adhesion molecules [19]. So, although HSE cell population is not homogeneous and includes EC1 and EC2, we analyzed the overall adhesive potential of B16M cells in the liver by pooling the two populations under a pro-metastatic inflammatory challenge. We used our *in vitro* organ-specific model of hepatic microvascular response to *C. albicans* to study the effect of different strains and morphologies of this fungus on B16M adhesion to HSE cells. Live blastoconidia of *C. albicans* strains, except CA2, began germination by 1 h after being added to HSE cells, and by 8 h after infection most of the endothelial cells were in contact with *Candida* germ tubes with no evidence of alteration in endothelial cell viability. All of the fungal cells were, then, easily removed from the cultures by extensively washing and it was checked by microscopy that none remained on the surface of the endothelial cells.

**Figure 1:**
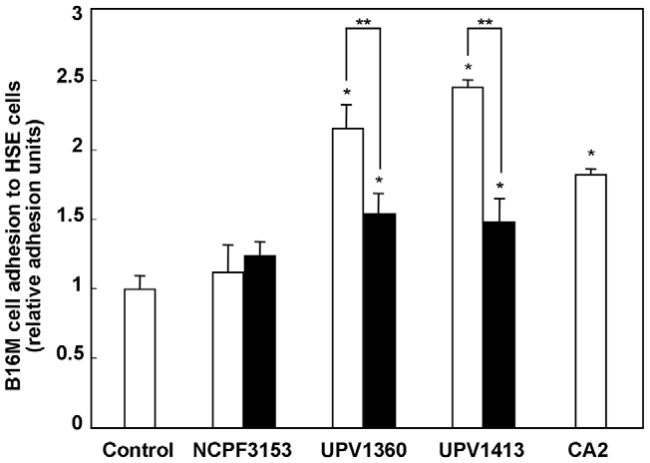
Effect of morphology of the different strains of *Candida albicans* on the increase in B16 melanoma (B16M) cell adhesion to hepatic sinusoidal endothelial (HSE) cells. Blastoconidia (□) and germ tubes (▪). The results shown correspond to the mean ± SD of three independent experiments. Statistically significant differences with respect to the control or between different morphologies are indicated by one (*) and two asterisks (**) (p<0.05), respectively.

As shown in Fig. 1 the adhesion of B16M cells to HSE cells significantly increased (p<0.05) with respect to the control when they had been inoculated with UPV1360 and UPV1413 clinical strains of blastoconidia, but not when the NCPF3153 reference strain was used. In addition, the differences between the increase induced by clinical strains and the reference strain were also significant.

As for the effect of the morphology, both forms of the clinical strains enhance tumor cell adhesion, but the increase was 53–67% lower (p<0.05) with germ tubes than with blastoconidia (Fig. 1). Interestingly, incubation of HSE cells with blastoconidia of a germination-deficient strain (CA2) also significantly increased (p<0.05) B16M cell adhesion compared to the control and to the reference strain NCPF3153 (Fig. 1).

### Study of the carbohydrate and protein composition in the crude extracts of the *C. albicans* strains

**Figure 2:**
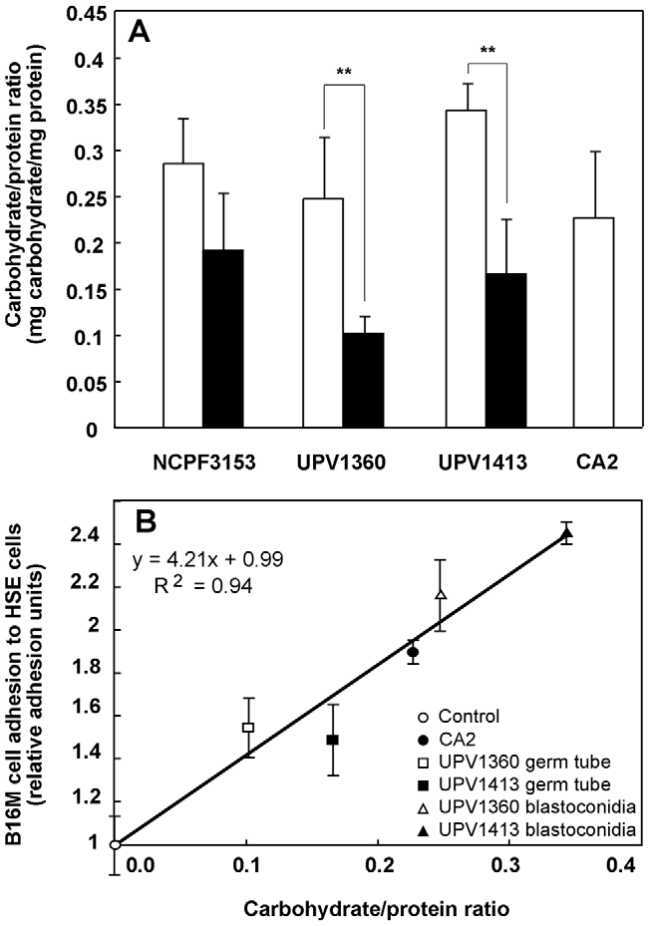
Study of the carbohydrate/protein composition in different strains and morphologies. (A) Carbohydrate/protein ratio in the crude extracts of the different strains of *Candida albicans*: Blastoconidia (□) and germ tubes (▪). The results shown correspond to the mean ± SD of three independent experiments. Statistically significant differences between different morphologies are indicated by two asterisks (**) (p<0.05). (B). Regression line for the B16 melanoma (B16M) cell adhesion to hepatic sinusoidal endothelial (HSE) cells induced by the strains and morphologies of *C. albicans* that lead a significant effect *versus* their carbohydrate/protein ratio.

Differences in the carbohydrate/protein ratio between strains could provide an explanation of which characteristics of *C. albicans* are primarily responsible for its effect on tumor cell adhesion to HSE cells. We measured the carbohydrate and protein concentrations of crude extracts from each strain in blastoconidia and germ tubes. The protein concentration was higher in germ tube than in blastoconidial extracts, while the carbohydrate concentration was similar (data not shown). This means that the carbohydrate/protein ratio is higher in blastoconidia than in germ tubes. Interestingly, this difference between blastoconidia and germ tubes was significant in the UPV1360 and UPV1413 strains (p<0.05), which induce the greatest effect on tumor cell adhesion to endothelial cells (Fig. 2 A). In fact, the plot of B16M cell adhesion to HSE cells *versus* carbohydrate/protein ratio of the strains and morphologies that induce a significant increase in tumor cell adhesion showed a linear relationship (R^2^ = 0.94) (Fig. 2 B).

### Effect of the viability of *C. albicans* on tumor cell adhesion to HSE cells

In order to determine whether the adhesion induced by *C. albicans* was dependent on cell viability and to confirm that surface carbohydrates were involved in the process, blastoconidia were killed either using heat or sodium metaperiodate.

**Figure 3:**
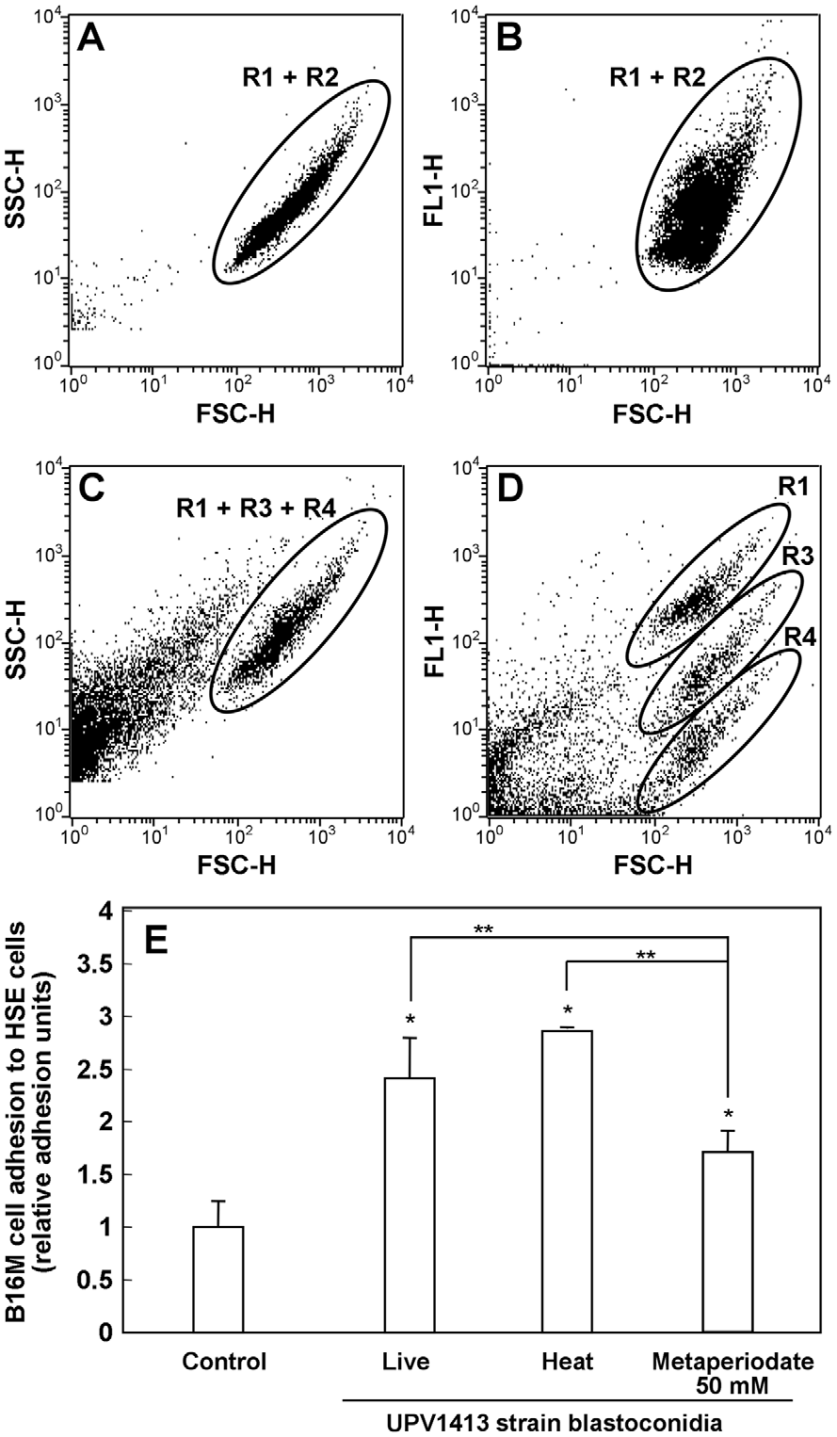
Results obtained from the killing treatments. (A–D) Effect of heat and metaperiodate treatments on *Candida albicans* cell surface studied by incubation with Con A-FITC followed by flow cytometry: cell complexity (side scatter, SSC), cell size (Forward Scatter, FSC), and fluorescence (FL1). R1: live cells. R2: cells killed with heat. R3: cells killed with a 20 mM metaperiodate. R4: cells killed with 50 mM metaperiodate. (E) Effect of heat- and sodium metaperiodate (50 mM)-treated *C. albicans* blastoconidia on the increase of B16 melanoma (B16M) cell adhesion to HSE. The results shown correspond to the mean ± SD of three independent experiments. Statistically significant differences with respect to the control or between cell conditions are indicated by one and two asterisks (**) (p<0.05), respectively.

Using flow cytometry, we demonstrated that neither the heat nor the metaperiodate treatments altered cell size or complexity (Fig. 3 A, C). On the other hand, while the heat treatment did not alter the binding capacity of Con A enough to distinguish live from dead cells, treatments with metaperiodate did inhibit the binding capacity of Con A to mannosylated molecules (attributable to mannose ligand residues having been destroyed on the cell surface) and both 30 and 50 mM-treated populations could be distinguished from live cells. We also found that 50 mM of metaperiodate was necessary to remove most of the carbohydrate (Fig. 3 D). This treatment significantly reduces (p<0.05) tumor cell adhesion to HSE cells, compared with the live cells. However, while the level of adhesion was slightly higher in those which underwent heat treatment, differences were not significant. In addition, it is notable that comparing data from the two treatments, metaperiodate-treated cells had approximately a 50% lower capacity to increase tumor cell adhesion than heat-treated ones, the difference being statistically significant (Fig. 3 E).

### Analysis of the differential expression of the MPF most relevant genes between different strains and morphologies of *C. albicans*

Differences in carbohydrate/protein ratio could not explain the lower effect of the reference strain in comparison with the clinical ones. Hence, we also studied the expression level of the 10 genes that code for the proteins identified with the best scores (>150) in the MPF that increases tumor cell adhesion to endothelial cells [14]. Samples of total RNA were collected from two clinical strains (UPV1413 and UPV1360) and a reference strain (NCPF3153), in both blastoconidia and germ tube morphologies, and in a non-germinating strain (CA2) and transformed into cDNA.

We designed the qPCR analysis (Table 1) to detect the expressions levels of each of the 10 genes: alcohol dehydrogenase (*ADH1*), aminopeptidase Y (*APE3*), isocitrate dehydrogenase subunit (*IDH2*), enolase (*ENO1*), fructose-bisphosphate aldolase (*FBA1*), ketol-acid reductoisomerase (*ILV5*), disulfide isomerase (*PDI1*), phosphoglycerate kinase (*PGK1*), ubiquinol-cytochrome-c reductase (*QCR2*) and translation elongation factor Tu (*TUF1*). We also used two genes, actin (*ACT1*) and glyceraldehyde 3-phosphate dehydrogenase (*TDH3*), as housekeeping genes to monitor the expressions levels. Both of these genes showed stable expression across all the different strains and morphologies studied.

**Figure 4:**
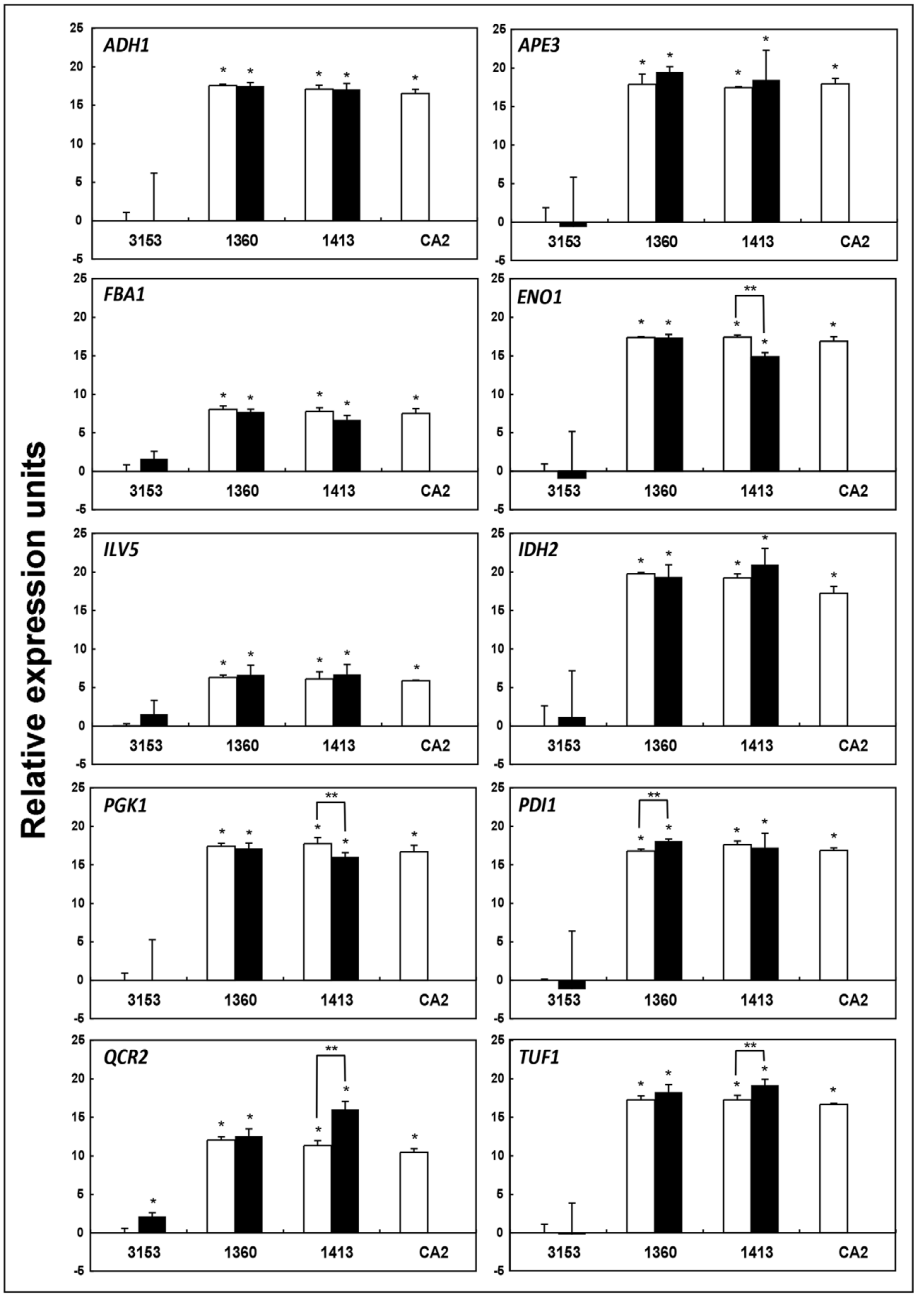
Differences in expression level of the genes identified in the pro-metastatic mannoprotein fraction. Expression level of the ten genes studied in different strains (NCPF3153, UPV1360, UPV1413, and CA2) and morphologies, blastoconidia (□) and germ tubes (▪), related to blastoconidia of *Candida albicans* NCPF3153 (control). Genes: alcohol dehydrogenase (*ADH1*), aminopeptidase Y (*APE3*), isocitrate dehydrogenase subunit (*IDH2*), enolase (*ENO1*), fructose-bisphosphate aldolase (*FBA1*), ketol-acid reductoisomerase (*ILV5*), disulfide isomerase (*PDI1*), phosphoglycerate kinase (*PGK1*), ubiquinol-cytochrome-c reductase (*QCR2*) and translation elongation factor Tu (*TUF1*). The results shown correspond to the mean ± SD of three independent experiments. Statistically significant differences with respect to the control or between different morphologies are indicated by one (*) and two asterisks (**) (p<0.05), respectively.

The qPCR conducted in this work enabled us to confirm that in all of the genes studied the transcription levels differed between the reference and clinical strains, being substantially higher in the latter (p<0.05) (Fig. 4). Moreover, we found a significantly higher level of transcription of these 10 genes in the CA2 non-germinating mutant strain than the wild reference strain NCPF3153.

The relative expression of these genes in the two morphologies was also assessed. We only found significantly different levels of expression in a few of the genes (p<0.05): *ENO1* and *PGK1* of UPV1413 were expressed more in blastoconidia than germ tubes; while, on the other hand, the expression of *QCR2* of NCPF3153, *PDI1* of UPV1360, and *TUF1* and *QCR2* of UPV1413 was significantly higher in germ tubes compared to blastoconidia (Fig. 4). In the other genes, there was no substantial difference in expression levels between morphologies of each strain.

### *C. albicans* induces tumor cell adhesion to HSE cells via the MR

**Figure 5:**
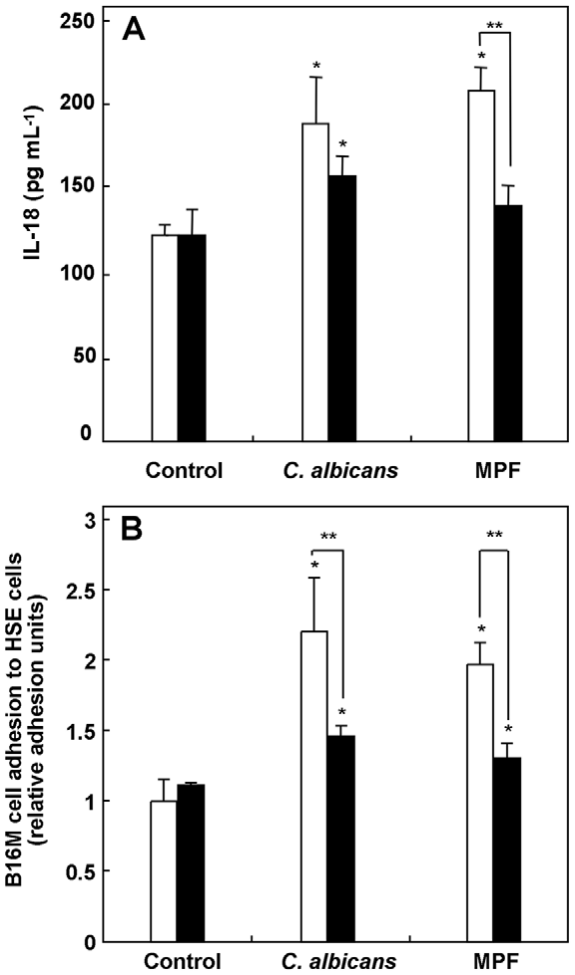
Effect of the mannose receptor (MR) inhibition. Effect of the anti-mannose receptor antibodies (anti-MR) on the inhibition of the synthesis of IL-18 (A) and tumor cell adhesion to hepatic sinusoidal endothelial (HSE) cells (B) induced by *Candida albicans* UPV1413 and its mannoprotein-enriched fraction (MPF): basal medium (□), 30 µg/ml anti-MR (▪). The results shown correspond to the mean ± SD of three independent experiments. Statistically significant differences with respect to the controls or to the cells without antibodies are indicated by one (*) and two asterisks (**) (p<0.05), respectively.

Given that the MPF seems to be the main factor responsible for the increase in tumor cell adhesion to HSE cells induced by *C. albicans* [14], the anti-MR antibody was used to assess the contribution of MR to the pro-inflammatory cytokine activation and B16M cell adhesion-stimulating effects of *C. albicans* and its mannoproteins on HSE cells. As shown in Fig. 5 A, the production of IL-18 increased in the presence of *C. albicans* cells (121.62 to 189.11 pg) and in the presence of MPF (121.62 to 208.10 pg), but after incubating HSE cells with anti-MR antibody the magnitude of these increases was reduced by 45.5% (121.62 to 158.43 pg) and 78.5% (121.62 to 140.21 pg) respectively. More importantly, treatment with the same antibody inhibited the increase in B16M cell adhesion induced by *C. albicans* blastoconidia and their MPF by 60.6% and 59.1%, respectively (Fig. 5 B).

## Discussion

In previous research [12], we showed that *C. albicans* favors the development of liver metastasis by endothelial cell activation that increases cytokine production and tumor cell adhesion. More recently, we have demonstrated *in vitro* that MPF was the main factor underlying this effect, and we have also identified some proteins, that could potentially, either together or separately, be responsible [14]. This can be understood in terms of the fact that the cytokine-cascade induced after endothelial stimulation increases the expression of adhesion molecules, such as VCAM-1, to which tumor cells can adhere [13], [19]. Although we checked that no fungal cell remained on endothelial cell surface after incubation, we cannot rule out that mannoproteins bind at the surface of the endothelial cells and constitute another ligand. However, it was demonstrated that the mannans were internalized by endothelial cells, after an exposure time of 1 h [20]. That is, we consider that the presence of mannoproteins as ligands is unlikely because the incubation time we used is long enough for endothelial cells to have internalized most of the bound mannoproteins.

Consistent with our observations, cancer patients have a higher risk of liver metastasis after a candidiasis, and this is attributable to the adhesion of the fungus or its mannoproteins to the hepatic endothelium. The ability of both morphologies, especially blastoconidia, of *C. albicans* to adhere to endothelial cells has been widely studied [1]–[4], as has the role of the different cell receptors of the innate immune system in the recognition of the microorganisms [5], [6] and, in some cases, even in the modulation of the immune response by different pathways depending on the morphology of the fungus adhered [21]–[24].

The influence of the morphology of different strains of *C. albicans* on the effect on tumor cell adhesion to endothelial cells was explored using our *in vitro* organ-specific model. These results demonstrated that the blastoconidia of all the strains tested enhanced tumor cell adhesion significantly more than germ tubes. Moreover, we found that carbohydrate/protein ratio was also higher in blastoconidia than in germ tubes. This could have a substantial relevance on the process considering that graphical analysis of data indicated a direct relationship between the carbohydrate/protein ratio and the tumor cell adhesion induced by the pro-metastatic *C. albicans* strains. The decrease in carbohydrate/protein ratio in germ tubes was mainly due to their increase in protein but not in carbohydrate concentration, and this seemed to be related to a weaker effect on tumor cell adhesion. We should not forget that the number of strains used is relatively low, and we cannot rule out that one or some of the new proteins of the germ tubes have an inhibitory effect, issues that it might be interesting to explore. Overall, however, considering that MPF was the most stimulatory fraction [14] and taking into account all the results that we report in this manuscript, mannoproteins appear to be the main effectors in the process studied. This is consistent with previous results that, on the basis of proteomics analysis involving immunodetection with Con A-HRP, demonstrated that there is a higher degree of mannosylation in blastoconidia than germ tubes [25], and with other authors that related the mannosylation of *C. albicans* with a pro-inflammatory response [26]. Our latest results might not only explain the greater effect of blastoconidia on tumor cell adhesion mediated by pro-inflammatory cytokines, but also could be consistent with the observation that blastoconidia activate a Th1 immune response, whereas germ tubes preferably induce a non-inflammatory Th2 immune response [21].

The relevance of mannoproteins in the process studied was also made visible by killing blastoconidia with heat and sodium metaperiodate treatments. The latter removed carbohydrates, including mannose ligand residues, and significantly reduced the tumor cell adhesion induced by *C. albicans*, whereas the heat treatment even slightly increased the effect with respect to the live cells. Specifically, comparing the results of the two killing methods, sodium metaperiodate-treatment reduced the adhesion induced to approximately 50% less than in heat-treated cells. The fact that killed blastoconidia and the CA2 germination deficient strain increased tumor cell adhesion seems to be inconsistent with reports of other authors that only live and germinated *C. albicans* induce cytokine expression by endothelial cells [7], [8], but results cannot be compared since the endothelial cells and *in vitro* model used were different. It would be interesting to study the effect of viable cells without carbohydrates on the surface, for example, after enzyme treatment, but the duration of the incubation with the endothelial cells (8 h) would be long enough to allow *C. albicans* to modify the fungal cell wall again. On the other hand, our previous results [14], and those reported in this manuscript, provide evidence that the increase in *C. albicans*-induced B16M cell adhesion is mainly due to the effect of mannoproteins, which could be found in the blood stream on live or killed blastoconidia, or alone, after secretion or release from disrupted cells.

In the same experiment with different strains of *C. albicans* using our *in vitro* organ-specific model of hepatic response against *C. albicans* we found that the two clinical strains used here have a greater effect on B16M cell adhesion to HSE than the reference one. Considering our results, the differences between the effects of the different strains cannot be related to different carbohydrate/protein ratio between them. Moreover, the fact that the reference strain does not increase adhesion as much as clinical isolates is perhaps surprising, since the *C. albicans* cell wall is composed of mannoproteins even in repeatedly subcultured strains. However, we found that the carbohydrate/protein ratio and increase in tumor cell adhesion were not directly related in the reference strain. So, differences between reference and clinical strains might be a consequence of proteins being differentially expressed. To corroborate this hypothesis, we studied the expression of the proteins identified by our group in the MPF of *C. albicans* with the strongest pro-metastatic effect. We performed qPCR for the genes that code for the proteins identified with highest scores in the fraction [14].

A notable finding was that the expression levels of the 10 genes of the MPF, *ADH1*, *APE3*, *IDH2*, *ENO1*, *FBA1*, *ILV5*, *PDI1*, *PGK1*, *QCR2* and *TUF1*, were significantly higher in clinical strains than in the reference one. Our results agree with the findings of Hernando *et* *al*., [27] that the subculturing of *C. albicans* strains alters antigen expression, and are in accordance with our observation that the clinical strains of *C. albicans* induce a greater increase in tumor adhesion than the reference one. So, the higher rate of synthesis of the proteins found in the MPF in the clinical strains could be related to the increase in tumor cell adhesion induced by *C. albicans*. However, their involvement cannot be assured and the effect of these proteins individually must be investigated further in the future.

Attending to the results obtained so far, we believe that the connection between *C. albicans* and the increase in tumor cell adhesion can be thought of as consisting of two factors: fungal cell post-translational mannosylation, which is responsible for the greater adhesion induced by bastoconidia than germ tubes; and expression level of some proteins of the MPF, which is apparently related to differences between strains.

It is well known that the mannosylated components in the bloodstream of the host are rapidly eliminated due to the presence of the MR [28]. So, our results suggest an important role of this receptor in the pro-metastatic effect that we report. Therefore, we used a commercial anti-MR antibody to assess the contribution of the MR to the pro-inflammatory cytokine activation and B16M cell adhesion-stimulating effects of *C. albicans* and its mannoproteins on HSE cells. It was found that anti-MR antibodies significantly reduced the stimulating effects of *C. albicans* and of MPF on IL-18 secretion with respect to the control (45.5–78.5%) and B16M cell adhesion by as much as 60%, similar to the results obtained by removing carbohydrates with the metaperiodate treatment. These results confirm the importance of MR in the endothelial response against this yeast species. However, as our results show, the inhibition is not complete; indicating that other pathways or receptors may be involved in the same stimulation of the endothelium. These receptors could recognize other carbohydrates, proteins or even a protein portion of the mannoproteins.

Other authors have studied the importance of IL-18 in B16M adhesion to HSE cells. They reported that IL-18 neutralization reduced the number of hepatic metastatic foci by 75% and the metastatic volume by 80% *in vivo* and prevented B16M cell adhesion completely *in vitro* [13], [29], [30]. We consider that total inhibition of the cytokine could be controversial, but that specific inhibition of the binding of *C. albicans* to mannose receptor without inhibition of the entire cytokine-mediated response might be helpful to control both cancer and infection without causing severe immunosuppression. Our findings have clinical implications as they suggest that the use of the anti-MR antibodies in therapies may help to reduce tumor invasiveness induced by *C. albicans* or its mannoproteins. Such antibodies might even be useful in combination with antifungal treatments to avoid the potential effect on tumor cell adhesion of molecules derived from destruction of the yeast, though this needs to be explored further in future research.

## Conclusion

*C. albicans* and its mannoproteins activate HSE mainly through MR and, in consequence, enhance tumor cell adhesion to endothelium by a pro-inflammatory mechanism. The degree of mannosylation of the fungal cells, and the expression level of the genes that code for, at least, the most abundant proteins of the MNF seem to be related to the pro-metastatic effect. These characteristics make blastoconidia of clinical *C. albicans* strains, regardless of their viability, the most dangerous form for the development of metastasis in liver. These findings may also be the basis of a new treatment for solid organ cancers based on the role of the MR in the studied *C. albicans*-induced tumor progression and metastasis.
